# The role of soy and soy isoflavones on women's fertility and related outcomes: an update

**DOI:** 10.1017/jns.2022.15

**Published:** 2022-03-07

**Authors:** Gianluca Rizzo, Alessandra Feraco, Maximilian Andreas Storz, Mauro Lombardo

**Affiliations:** 1 Independent Researcher, Via Venezuela 66, 98121 Messina, Italy; 2 Laboratory of Cardiovascular Endocrinology, IRCCS San Raffaele Roma, 00166 Rome, Italy; 3 Department of Human Sciences and Promotion of the Quality of Life, San Raffaele Roma Open University, 00166 Rome, Italy; 4 Center for Complementary Medicine, Department of Internal Medicine II, Faculty of Medicine, University of Freiburg, Freiburg, Germany

**Keywords:** Assisted, Fecundability, Fertility, Infertility, Isoflavones, Phytoestrogens, Reproductive techniques, Soy, Soybeans, Soy foods

## Abstract

Soy is a key food in human nutrition. It is widely used in eastern traditional cuisine and it has recently diffused among self-conscious and vegetarian diets. The success of soy mainly depends on versatility and supposed healthy properties of soy foods and soy components. Meanwhile, the possible influence on endocrine system, in particular by isoflavones, raised concerns among some researchers. The present paper aims to conduct a review of available data on the effect of soy, soy foods and soy components on women's fertility and related outcomes. Eleven interventional studies, eleven observational studies and one meta-analysis have been selected from the results of queries. A weak, not clinically relevant effect has been highlighted on cycle length and hormonal status. However, a suggestive positive influence has been shown among women with fertility issues and during assisted reproductive technologies. Overall, soy and soy components consumption do not seem to perturb healthy women's fertility and can have a favourable effect among subjects seeking pregnancy. However, because of the paucity of studies exploring the impact of soy intake on women's fertility, as well as the limited population sample size, the frequently incomplete specimens’ collection to investigate all cycle phases and the insufficient characterisation of participants, the evidence is suggestive and it needs further in-depth research taking into account all these aspects.

## Graphical abstract

**Figure:**
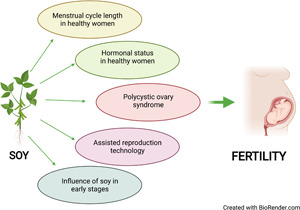

## Introduction

Soy is a very popular food and its consumption is part of the traditional cuisine of South-East Asian countries. However, even in the West, it is currently widely used, especially due to its versatility in plant-based products for health purposes and vegetarian diets^(1)^. Interest in soy is particularly driven by its possible beneficial effects on human health. Soy consumption is supposed to have protective effects against cardiovascular disease by cholesterol-lowering and blood pressure improvement action and in the prevention of cancer or diabetes and it also supports bone health and the management of menopause symptoms^(2–8)^.

Soy contains numerous phytochemicals that can be responsible for these positive effects through multiple mechanisms. Many of its components show an antioxidant activity that can at least partially explain its effectiveness^(9)^. In particular, soy contains numerous non-isoflavone constituents such as phytic acid, triterpenes and sterols, Bowman–Birk protease inhibitors, unsaturated fatty acids, saponins, inositol phosphates, proteins, peptides such as lunasin;^(10)^ nevertheless, soy isoflavones have attracted much attention in the last years for its estrogenic as well as non-hormonal properties^(11)^.

Isoflavones are non-steroidal compounds with a chemical structure similar to endogenous estrogens and for this reason, they are defined as phytoestrogens: a functional classification that also includes lignans, coumestans and stilbenes^(12)^. Notably, these latter compounds are present in several foods such as legumes, cereals and seeds, whereas soy is almost the only source of isoflavones in human diet. Isoflavones show several biological properties, acting as selective tissue estrogenic activity regulators (STEARs), thanks to the differential distribution pattern of estrogen receptors in body tissues^(13)^ and the differentiated affinity between the two isoforms of estrogen receptors, called alpha and beta. Moreover, isoflavones act as selective estrogen receptor modulators (SERMs) showing both agonist and antagonist effects on ER, with subsequent estrogenic, anti-estrogenic or even neutral effects^(14)^.

Regarding isoflavones, the equol metabolite derives from the precursor daidzein by the action of intestinal bacteria. The individual conversion capacity, equol-competence, offers a useful tool for estimating the biological effect of these compounds^(15)^. Individuals who are not equol-producers have likely limited response to isoflavone intake^(16)^. Even if the exact conversion mechanism has not been characterised yet, a limited conversion capacity in Western populations (about 25 %) has been highlighted, as opposed to the greater competence of Asian populations (50 %), estimated through urinary equol excretion^(17)^. For this reason, in clinical studies, the nationality and ethnicity of participants may be relevant for the assessment of potential conflicting effects of soy intake.

Soy isoflavones seem to act also through a non-genomic regulation, activating specific cellular signalling pathways^(18)^. Finally, they show antioxidant activity: a shared property among polyphenols^(19)^. The beneficial efficacy of soy is often attributed to the presence of isoflavones, capable of mitigating the excesses of endogenous estrogens, through the competition with estrogen receptors or by the activation of receptors, in the presence of low levels of endogenous estrogens. On the other hand, many perplexities have been raised about possible negative mechanisms leading to endocrine disruptor effects^(20)^.

Consequently, it is plausible that research efforts have been aimed at evaluating the effects of soy, especially isoflavones, on human fertility and hormonal regulation. As for males, a 2010 meta-analysis highlighted the safety of soy on fertility outcomes^(21)^, recently confirmed by an updated meta-analysis on this topic^(22)^. However, the specific effect of soy intake on women's fertility has not yet been systematically evaluated. The purpose of this review is to collect currently available data in literature, summarising the possible interaction between soy, soy foods and components of soy (in particular isoflavones) on aspects concerning women's fertility and related outcomes.

## Methods

A systematic consultation of literature was launched on four search engines (PubMed, ScienceDirect, Cochrane Trials Library and ClinicalTrials.gov) using the following keywords:

(‘Soy’ OR ‘Soy Foods’ OR ‘Soybeans’ OR ‘Genistein’ OR ‘Daidzein’ OR ‘Isoflavones’ OR ‘Phytoestrogens’) AND (‘Fertility’ OR ‘Infertility’ OR ‘Fecundability’).

Keywords were searched in titles and abstracts and combined with MeSH terms, where available, adapting the query format based on the search engine used. No restrictions were applied using filters and results were collected from search engines by the inception through 4 April 2021. The obtained results were evaluated for duplicates and then screened for titles and abstracts information. For the remaining papers, the full texts were retrieved for the final evaluation and inclusion in the summary.

Articles concerning reviews, case series, case studies, non-human studies, *in vitro* studies, studies on males, editorials, letters to editor, conference abstracts, book's chapters, non-English papers, studies with no-soy isoflavones and studies with outcomes not pertinent to fertility were excluded. In addition, full-text bibliographic lists from selected papers were screened to retrieve further relevant articles. The procedure was carried out following the most recent PRISMA guidelines^(23)^.

## Results and discussion

**Fig. 1.**
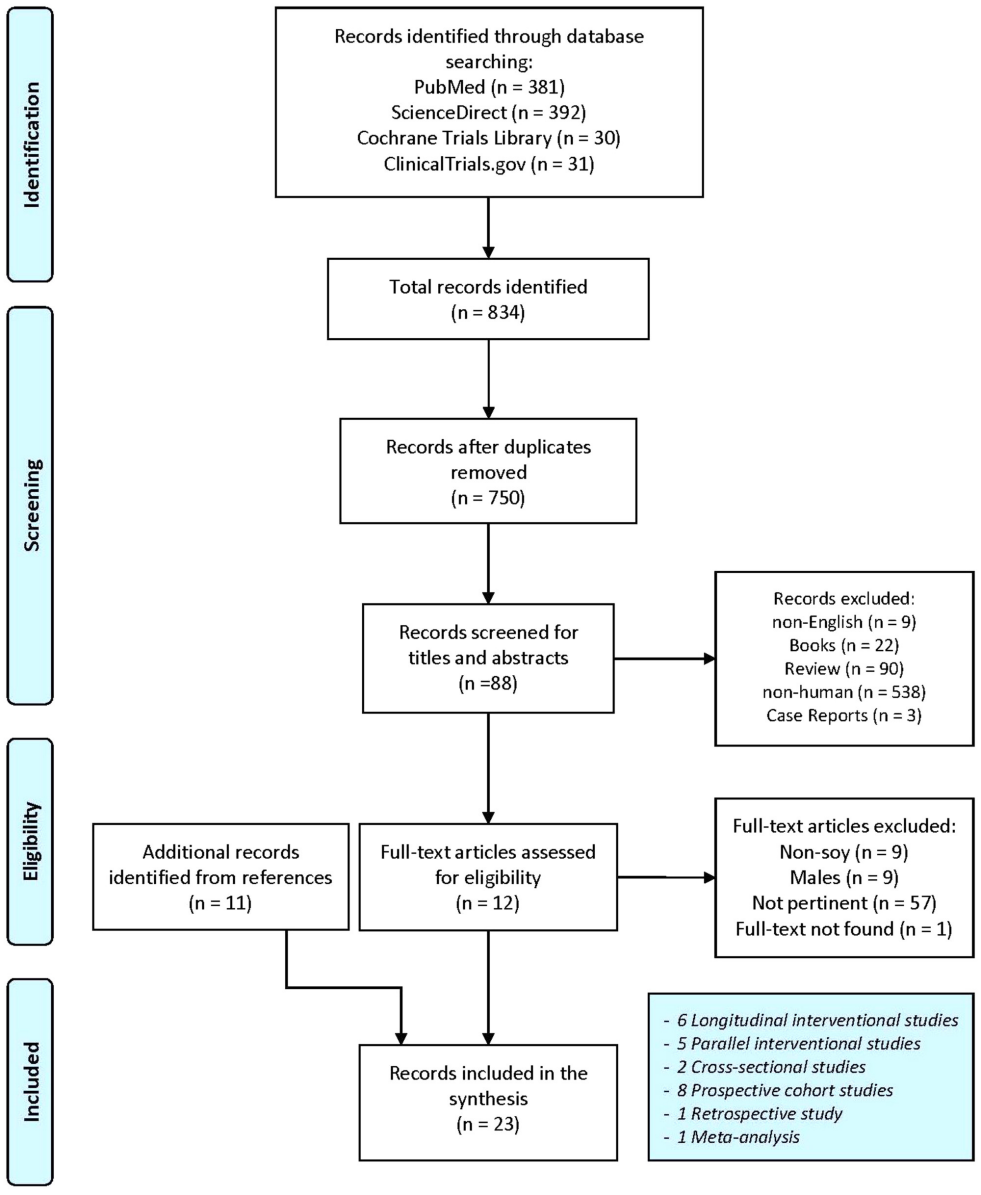
Flowchart for studies selection. Adapted from Moher *et al.*^(24)^.

A total of 834 entries were obtained following search engine queries (PubMed: 381; ScienceDirect: 392; Cochrane Library Trials: 30 and ClinicalTrials.gov: 31). Following the removal of eighty-four duplicates, the selection was made through titles, abstracts and full-text reading. Finally, twelve entries were identified and ten additional articles were obtained after the consultation of full-text bibliographic lists. A total number of twenty-two experimental articles plus a meta-analysis was used for the final synthesis. The detailed selection process is highlighted in Fig. 1.

The obtained meta-analysis was included in the final summary because it assessed aspects relating to the topic of this review. This allowed to exclude the already summarised articles in meta-analysis from a detailed discussion. The results of selected manuscripts were grouped according to the outcomes used, for a clear comparison. Manuscripts exploring multiple aspects were discussed in different paragraphs, where deemed necessary.

The clinical studies selection included one retrospective study, two cross-sectional studies, eight longitudinal cohort studies, five parallel-designed interventional studies and six longitudinal interventional studies. A list of the selected clinical studies with their characteristics is summarised in Table 1.

**Table 1.** Main characteristics of selected studies

Reference	Topic	Study design	Country/Ethnicity	*n*	Participants	Intervention	Duration	Main results
Petrakis *et al.*^(25)^	Hormonal status	Longitudinal interventional	USA/Caucasian, African-American	14	Healthy	37⋅4 g/d soy protein isolate (37⋅4 mg Gen)	6 months	Higher serum E2 levels during soy intervention phase were shown compared with no-soy phases. No changes in progesterone, LH or SHBG after soy intervention
Lu *et al.*^(26)^	Menstrual cycle length Hormonal status	Longitudinal interventional	USA/Caucasian, Hispanic, African-American	6	Healthy	36 Oz/d soy milk (~200 mg/d IF)	1 month	Serum E2 (31 % at days 5–7, *P* = 0⋅09; 81 % at days 12–14, *P* = 0⋅03; 49 % at days 20–22, *P* = 0⋅02) and *P* levels of luteal phase (35 %, *P* = 0⋅002) decreased after soy intervention compared with baseline. DHEAS levels were reduced after soy intervention (23 %, *P* = 0⋅03). The menstrual cycle was marginally increased by intervention (from 28⋅3 ± 1⋅9 to 31⋅8 ± 5⋅1 d, *P* = 0⋅06)
Nagata *et al.*^(27)^	Hormonal status	Cross-sectional	Japan/Japanese	50	Healthy	–	–	E2 levels were inversely associated with soy intake, assessed with multiple adjusted spearman rank correlation coefficients. Miso intake was inversely associated with SHBG levels. No association between soy and cycle length
Wu *et al.*^(28)^	Menstrual cycle length Hormonal status	Longitudinal interventional	USA/Caucasian, Hispanic, Asian	20	Healthy	Soy foods (32 mg IF/d)	7 months	Reduced E2 levels in follicular phase but not in the luteal phase. *P* and SHGB level were not significantly changed. Reduced serum E2 levels among Asian but not for non-Asian participants after intervention, compared with baseline. Influences on E2 level were attenuated using ‘clean’ dataset. No significant association for soy intake with the cycle length was shown
Lu *et al.*^(29)^	Menstrual cycle length Hormonal status	Longitudinal interventional	USA/Caucasian, African-American	10	Healthy	36 Oz/d soy drink (113–207 mg/d IF)	1 menstrual cycle	Reduced serum E2 (23 %, *P* < 0⋅01) and *P* levels (45 %, *P* < 0⋅0001) with soy intervention compared with baseline were highlighted. No significant variation in cycle length, LH and FSH concentrations. Serum or urinary genistein and daidzein were associated with luteal phase length reduction in multiple regression model but not with *P* levels
Strom *et al.*^(30)^	Early exposure	Retrospective	USA/mainly Caucasian	128: soy formula, 268: cow formula	Soy/cow formula	–	–	No significant differences in fertility outcomes such as missed menstrual periods, pregnancy, live births, abortions, miscarriages, full-term deliveries, preterm deliveries, between soy or cow formula utilisers in infancy were seen
Unfer *et al.*^(31)^	Assisted reproduction	Parallel Interventional	Italy/Caucasian	65: intervention, 69: placebo	Fertility issues	1⋅5 g/d Ph	10 d	Improved endometrial thickness using transvaginal sonography in soy group compared with placebo. The soy group showed lower rates of miscarriage (*n*: 2, 3⋅1 % *v*. *n*: 6, 8⋅7 %; *P* < 0⋅05) and higher rates of pregnancy (*n*: 13, 20⋅0 % *v*. *n*: 3, 4⋅4 %; *P* < 0⋅05) compared with placebo. No significant differences for follicular development, the number of preovulatory follicles and the pulsatility index values
Unfer *et al.*^(32)^	Assisted reproduction	Parallel Interventional	Italy/Caucasian	115: intervention; 98: placebo	Fertility issues	1⋅5 g/d Ph	At least one cycle of treatment	Increased implantation rate (25⋅4 % *v*. 20⋅2 %; *P* < 0⋅05), pregnancy rate (39⋅3 % *v*. 20⋅9 %; *P* < 0⋅05) and pregnancy-to-delivery rate (30⋅3 % *v*. 16⋅2 %; *P* < 0⋅05) in soy group compared with placebo
Kohama *et al.*^(33)^	Fertility Hormonal status	Parallel Interventional	Japan/Asian	36: intervention; 34: control	Secondary amenorrhea or anovulation	6 g/d black soy extract	6 months	Fertility outcomes improvements (four pregnancies and twelve ovulations) compared with the control group (zero pregnancies, two ovulations), *P* < 0⋅05. Higher serum estradiol levels in soy intervention compared with baseline were highlighted
Romualdi *et al.*^(34)^	PCOS Hormonal status	Longitudinal interventional	Italy/Caucasian	12	PCOS	36 mg/d Gen	6 months	No hormonal changes (E2, FSH, LH, SHBG, Testosterone, DHEAS, Androstenedione, hydroxyprogesterone) after isoflavone intervention compared with baseline
Khani *et al.*^(35)^	PCOS Hormonal status	Parallel Interventional	Iran/Indo-European	69: intervention, 68: placebo	PCOS	36 mg/d Gen	3 months	The intervention with genistein reduced serum LH, DHEAS and testosterone levels compared with baseline
Jarrell *et al.*^(36)^	Fertility	Prospective Cohort	Canada/–	323	Late pregnancy	–	From second trimester until delivery	No association in Spearman's test correlation analysis between soy intake or amniotic Ph and self-reported infertility or pregnancy complications (premature labour, gestational diabetes, pregnancy-induced hypertension, low birth weight, caesarian section) among participants
Filiberto *et al.*^(37)^	Hormonal status	Prospective Cohort	USA/Caucasian, African-American, Asian, Other	259	Healthy	–	Up to two menstrual cycles	No association in adjusted linear mixed model between IF intake and ovulatory functions (serum E2, free E2, P, LH, FSH levels and sporadic anovulation assessed by *P* and LH levels) except for higher serum levels of SHBG (*β*: 0⋅09, 95 % CI 0⋅02, 0⋅16)
Jacobsen *et al.*^(38)^	Fertility	Cross-sectional	USA – Canada/Caucasian, African-American	11 688	Healthy Adventists	–	–	An inverse relationship between dietary IF intake and live birth (3 % reduction, 95 % CI 0, 7) and a positive relationship with nulligravidity were seen (13 % higher risk, 95 % CI 2, 26)
Mumford *et al.*^(39)^	Fertility	Prospective Cohort	USA/Caucasian, African-American, Hispanic, Others	471	Healthy, seeking for pregnancy	–	12 months or until pregnancy	No association between urinary IF and fertility, assessed by time to pregnancy
Vanegas *et al.*^(40)^	Assisted reproduction	Prospective Cohort	USA/Caucasian, African-American, Hispanic, Asian	315	Fertility issues	–	At least one cycle of treatment	Amelioration of clinical outcomes for *in vitro* assisted reproduction among women consuming soy compared with non-consumers, assessed with fertilisation rate (77 % *v*. 71 %, *P* = 0⋅004), age-adjusted clinical pregnancy rate (52 % *v*. 41 %, *P* = 0⋅03) and live birth rate (44 % *v*. 31 %, *P* = 0⋅007). In the second and third tertiles of IF consumption, higher live birth odds were seen in multivariable-adjusted analysis (OR: 1⋅87; 95 % CI 1⋅12, 3⋅14; OR: 1⋅77; 95 % CI 1⋅03, 3⋅03; respectively). Soy consumption was not related to estradiol levels or endometrial thickness
Andrews *et al.*^(41)^	Menstrual cycle length	Prospective Cohort	USA/Caucasian, African-American, Asian, other	246	Healthy	–	One or two menstrual cycles	Marginal reduction of luteal phase for an increase of 10 mg/d IF intake in the adjusted model (aOR: 1⋅38, 95 % CI 0⋅99, 1⋅92, *P* = 0⋅06), and significant for peak luteal progesterone ≤10 ng/ml (aOR: 1⋅40, 95 % CI 1⋅00, 1⋅96, *P* = 0⋅05), assessed with monitoring of LH levels
Chavarro *et al.*^(42)^	Assisted reproduction	Prospective Cohort	USA/Caucasian, African-American, Asian, other	239	Fertility issues	–	At least one cycle of treatment	Increased fertility (assessed with live birth rates, implantation and clinical pregnancy rates) with higher soy intake through interaction with urinary BPA, compared with no-soy consumption
Jamilian *et al.*^(43)^	PCOS Hormonal status	Parallel Interventional	Iran/Indo-European	35: intervention, 35: placebo	PCOS	50 mg/d IF	12 weeks	Soy IF intake reduced the free androgen index (−0⋅02 ± 0⋅005 *v*. +0⋅01 ± 0⋅005, *P* < 0⋅001), total testosterone (−0⋅1 ± 0⋅05 *v*. +0⋅1 ± 0⋅05 ng/ml, *P* = 0⋅008), and increased SHBG (+4⋅0 ± 0⋅8 *v*. −1⋅4 ± 0⋅8 nmol/l, *P* < 0⋅001) compared with placebo
Wesselink *et al.*^(44)^	Fertility	Prospective Cohort	USA – Canada – Denmark/Caucasian, African-American, Asian, Hispanic, others	7778	Healthy, seeking for pregnancy	–	1 year or until pregnancy	No association between IF intake and fertility (assessed by per-cycle probability of conception) with some marginal evidence of amelioration over 30 years associated with IF
Levine *et al.*^(45)^	Menstrual cycle length	Prospective Cohort	USA/Caucasian, African-American, Hispanic, other	326	Healthy	–	1 year or until pregnancy	Urinary Ph levels were inversely associated in adjusted regression models with cycle length; urinary Gen levels were associated with cycle irregularity (assessed by fertility monitors and daily journals)
Haudum *et al.*^(46)^	PCOS	Longitudinal interventional	Austria/Caucasian	24: PCOS, 20: healthy control	PCOS or Healthy	400 ml/d soy drink (50 mg IF)	3 d	Fertility amelioration (based on androgens and AMH levels) among equol-producers in the whole cohort compared with non equol-producers. No changes in DHEA, DHEAS, dihydrotestosterone (DHT) concentrations or LH:FSH ratio

### Fertility

Fertility is defined by the number of offspring produced by an individual. Consequently, the absence of fertility, called infertility, is a disease characterised by the failure to establish a clinical pregnancy and it can depend on various factors including predisposition and behavioural/environmental aspects. The fertility concept differs from the fecundity one, which refers to reproductive potential and depends on reproductive physiology, from the production of gametes phase to the ability to carry a pregnancy to term. Therefore, the lack of fecundity is called sterility^(47)^. However, the terms are often interchangeably, being closely associated with the possibility of giving birth to children. Recently, the anti-Müllerian hormone (AMH) concentrations have proved to be a useful tool for predicting female fertility, especially because it is independent of the cycle phase^(48)^.

In 2005, Kohama and colleagues published a short communication about a 6 months clinical trial on thirty-six Japanese women with secondary amenorrhea (or anovulation)^(33)^. Six grams per day of black soybean powder were administered to the intervention group, whereas thirty-four individuals received no treatment as a control group. After the intervention period, four patients became pregnant and twelve patients showed ovulation improvements based on ultrasonography (*P* < 0⋅05). Conversely, the improvements in ovulation were seen only in two patients from the control group. After 6 months, estradiol levels of patients in the intervention group were higher compared with basal (*P* < 0⋅05), whereas luteinizing hormone (LH) and follicle-stimulating hormone (FSH) were unchanged.

Black soy, in addition to the content of isoflavones, is known to be rich in antioxidant substances, especially in external seed integuments, which are rich in anthocyanins^(49)^. These substances could play a role in the ovaries circulatory functions^(50)^. Unfortunately, the work of Kohama *et al.* was a pilot study without a characterisation of diet among participants and without data on soy composition (isoflavone or antioxidant contents). It does not appear to be randomised and blinded, but the nature of outcomes should not be affected by these limitations.

In addition to the interventional study by Kohama and colleagues, we found three longitudinal cohort observational studies^(36,39,44)^ and a cross-sectional study^(38)^ that investigated the association between soy and fertility. Jacobsen and colleagues included 11 688 American women aged 30–50 years of age who participated in Adventist Health Study-2 (AHS-2)^(38)^. The Adventist Church is a community with very homogeneous habits and a high prevalence of vegetarians (54 % lacto-ovo vegetarian and 7 % vegan from this study)^(51,52)^.

Based on this cross-sectional study, high consumption of soy isoflavones was identified (94 % of participants). The significant inverse association between dietary isoflavone intake and live births (3 % reduction, 95 % CI 0, 7, *P* = 0⋅05) was highlighted. Similarly, a significant correlation between isoflavone intake and nulligravidity emerged (*P* = 0⋅03) with a 13 % higher risk but with a wide range of confidence interval (95 % CI 2, 26) in women with intake ≥40 mg/d compared with lower intakes (<10 mg/d). Only 6 % of participants had not soy isoflavone intake. There was no relationship between isoflavone intake and reported problems becoming pregnant. The generalisation of these results is complex due to the type of study, which does not allow to define a causal relationship. The consumption of soy over time, the possible use of certain foods considered healthy in seeking pregnancy or the willingness to avoid pregnancy could generate spurious associations.

The use of surveys only through self-administered questionnaires, although validated, is easily exposed to self-reporting errors or incompleteness and misclassifications derived from the database used for food intake quantification. Furthermore, the nutritional habits of Adventists differ from the Western population ones and they show soy consumption more similar to populations in South-East Asia^(53)^.

Among selected prospective cohort studies, in 2012 Jarrell and colleagues conducted an observational study of 323 Canadian women with late pregnancy (aged at least 35) followed from the second month of pregnancy until delivery^(36)^. The concentration of isoflavones in the amniotic fluid was related to soy intake, but there was no significant association between soy intake or phytoestrogens in the amniotic fluid and complications of pregnancy or previous infertility. Higher soy products intake did not correlate with the rate of infertility. However, only 106 individuals provided information on soy intake. Furthermore, the type of dietary survey carried out in the proposed environmental questionnaire was not clear. The same authors admitted that they had no information on the type of soy used and about the last ingestion. It would have been useful to have retrospective information on soy consumption to assess the potential effect on previous fertility problems. These aspects were poorly characterised by self-reporting of the participants. Other weak aspects of the population sample characterisation are given by a lack of stratification by ethnicity and equol-producers.

In another prospective study, 471 healthy American women were followed for 12 months or until delivery without showing significant correlations between urinary isoflavones, quantified by HPLC-MS analysis, and fertility, defined with adjusted Cox Model using time-to-pregnancy assessment^(39)^, while lignan concentrations in urine were significantly associated with shorter time to pregnancy. The study involved a large number of couples seeking pregnancy. In order to assess the association between urinary isoflavones and fertility, adjustment for various confounding factors including ethnicity, supplement use, nutrients and lifestyle aspects was applied. Measurement of urinary isoflavones and their metabolites appears to be a more reliable approach than dietary assessment alone. However, the intake of isoflavones in diet has not been investigated, and therefore, it was not possible to define the presence of equol-producers among participants. Moreover, urinary concentrations seem to reflect the isoflavone intakes in a short time window. The present study used a community-based approach with recruitment of couples seeking pregnancy. This could have introduced other confounding factors such as the influence of male on couple's fertility or possible changes in habits caused by the desire to conceive.

Recently, Wesselink and colleagues evaluated the fertility of 7778 healthy American or Canadian women in two cohort studies that followed participants for 12 months or until pregnancy^(44)^. The dietary intake of isoflavones did not appear to be associated with fertility in the two cohorts but some marginal evidence of amelioration of fertility was related to a higher intake of isoflavones among ≥30 years old individuals after age stratification (Fecundability Ratios: 1⋅12, 95 % CI 9⋅94, 1⋅34 and 1⋅19, 95 % CI 0⋅92, 1⋅55 in the two cohorts comparing ≥90th with <24th percentile). There was no dose-response relation in either cohort. Correction for covariates included demographics, education, income, lifestyle, dietary and behavioural factors. However, the intakes of isoflavones in the studied cohorts were limited (range: 0⋅3–3⋅1 mg/d). The FFQ was not specifically designed for phytoestrogen assessment and this may have underestimated intakes. Participants recruited were seeking for pregnancy and this could have been a source of confounders.

From the data obtained, diet isoflavones do not seem to have a direct effect on fertility, whether positive or negative. In the only clinical trial available, even if it is considered a pilot study, it emerges that a significant role could be played not only by isoflavones, but also by phytochemicals present in soy, particularly in black soy. This phenomenon highlights how in literature there is greater attention to phytoestrogens and their effect, frequently underestimating the role of other components that have a marginal interest.

### Menstrual cycle length in healthy women

The length of menstrual cycle may represent an indirect marker of ovarian function and reproductive health^(54,55)^. Fertility is closely associated with menstrual cycle functions and a longer time to pregnancy is associated with shorter menstrual cycles^(56–58)^.

Five studies exploring the relationship between soy and the length of menstrual cycle in healthy women have been selected, including two observational studies^(41,45)^ and three longitudinal interventional studies^(26,28,29)^.

With regards to available clinical trials, Lu and colleagues conducted two interventional studies using 36 Oz of soy milk (about one litre) divided into three daily intakes for a total daily intake of about 200 mg of isoflavones^(26,29)^. In the first study, the authors administered soy milk to six American women aged 22–29 for 1 month, comparing outcomes with baseline^(26)^. After the soy intervention, the length of menstrual cycle marginally increased (from 28⋅3 ± 1⋅9 to 31⋅8 ± 5⋅1 d, *P* = 0⋅06). The small number of participants significantly limited the quality of results. Furthermore, there was no characterisation of dietary regimen, although it was a standard hospital diet. Participants were classified by ethnicity; however, the population sample size did not allow to perform stratification of outcomes based on this aspect. No investigation into the individual's ability to absorb and use isoflavones from soy milk was performed. Besides, the lack of a placebo group warrants caution.

In the second study by Lu and colleagues^(29)^, ten American women aged 23–42 who did not consume soy regularly were followed for the duration of a menstrual cycle, during which nutritional intervention with soy was performed (36 Oz/d soy drink; 113–207 mg/d IF), without observing significant changes in cycle length compared to baseline and with a marginal shortening of luteal phase (6 %, *P* = 0⋅07). However, in multiple regression analysis, this reduction seemed to be significantly associated with the intake of genistein and daidzein or their concentration in urine. Similar to the previous trial, the number of participants was limited. The intervention period was extended only to one menstrual cycle. Furthermore, no characterisation was made on the possible presence of equol-producers among the participants. However, the sampling during the various days of the cycle allowed a detailed characterisation of serum LH surge day. Multiple regression analysis including various set of possible confounders highlighted more in-depth correlations. The evaluation of isoflavones circulating levels and their urinary excretion allowed to show a wide inter-individual variation of metabolic and absorption capacity.

In 2000, Wu *et al.* conducted a 7-month interventional study on twenty healthy American women aged 21–44, half of them of Asian origin, using various types of soy foods (soy milk, edamame, tofu) for an overall daily intake of about 32 mg of isoflavones^(28)^. Consistent with the previously cited data, no significant alteration in the cycle length was found among participants following the intervention. Additional considerations regarding hormonal influences will be discussed in the next paragraph.

Regarding observational studies, in 2015 Andrews and colleagues conducted a prospective cohort study on 246 American women with normal menstrual cycle, aged 18–44 and with 13 % of participants of Asian ethnicity, for a follow-up of 1–2 whole menstrual cycles^(41)^. The authors highlighted a marginal reduction of luteal phase in the adjusted multivariable model for an increase of 10 mg/d of dietary isoflavones (aOR: 1⋅38, 95 % CI 0⋅99, 1⋅92, *P* = 0⋅06), identified by monitoring LH levels in urine by a fertility monitor and 4-d per cycle 24-h dietary recalls. Similar significant association was observed for peak luteal progesterone ≤10 ng/ml (aOR: 1⋅40, 95 % CI 1⋅00, 1⋅96, *P* = 0⋅05). Corrections for confounding factors, such as diet, demographics, lifestyle factors, age, body composition and ethnicity, indicated reliable analysis. However, the evaluation of ability to absorb and metabolise isoflavones was lacking in the present study. This is justified by the fact that the study was not designed for the specific assessment of dietary soy concerning fertility-related outcomes. Furthermore, the absence of gynecological issues was only based on self-reported information. The study must be considered exploratory, because of the limited number of luteal phase deficiency cycles and a small number of fertility-related outcomes.

Recently, in a prospective study by Levine *et al.*, 326 American women eumenorrheic aged 18–40 were followed for 12 months or until pregnancy^(45)^. The authors showed an inverse correlation between cycle length (detected via fertility monitors and daily journals) and total urinary phytoestrogen levels (−0⋅042 d for 10 % increase, 95 % CI −0⋅080, −0⋅003). These also included non-soy derived phytoestrogens, such as lignans. No correlation with specific isoflavones such as equol, daidzein and O-DMA was found. Such shorter menstrual cycle length seemed not clinically relevant because shorter than 1 d. Furthermore, for each 1 nmol/l increase of genistein, the risk of menstrual cycle irregularities increased (OR: 1⋅19, 95 % CI 1⋅02, 1⋅38). The use of urinary phytoestrogens and their metabolites is a more reliable system compared to the evaluation of dietary intake. However, urinary phytoestrogen levels were only detected at baseline and this increased the correlation uncertainty. Despite adjustments for demographic, lifestyle, dietary factors, including ethnicity and other phytoestrogens, it would have been useful to check the dietary intake of isoflavones for equol-producers evaluation. Furthermore, the individuals recruited were seeking for a pregnancy and this could have changed their behaviour. Additionally, the enrolment criteria included only women who had stopped oral contraception less than 2 months earlier, so highly fertile individuals could have been excluded.

Luteal phase deficiency can represent a relevant aspect for pregnancy outcomes and fertility disorders. However, the association between soy and isoflavones with the reduction of luteal phase seems weak. The possible correlation between menstrual cycle length and soy does not seem convincing either.

In the meta-analysis by Hooper and colleagues^(59)^ from the evaluation of eleven studies on premenopausal women, ten studies were included to clarify the effect of soy on menstrual cycle length. A slight increase of approximately 1 d (MD: 1⋅05, 95 % CI 0⋅13, 1⋅97) was seen compared with the control, with no significant effects in the length of luteal and follicular phases. However, after removing data from studies with elevated bias risk, three studies were included in the sensitivity analysis with consequent loss of statistical significance.

Emerged clinical trials display several limitations including small sample size as well as the longitudinal design without a parallel control group, placebo or a cross-over design consistently limiting the strength of these pilot studies. Similarly, the duration of interventions is limited and equol-producers have not been identified. The two observational studies also show different limitations, in particular, one of these studies uses a follow-up of only 2 months. Pending further confirmation, soy and its components do not appear to have a clinically relevant influence on menstrual cycle in healthy women.

### Hormonal status in healthy women

Among the studies discussed to evaluate menstrual cycle length, three interventional studies also evaluated the levels of circulating hormones following soy intake in healthy women^(26,28,29)^. In the first of the two papers by Lu and colleagues^(26)^, the intake of 36 Oz/d of soy milk (~200 mg/d IF) for 1 month caused a reduction in mean estradiol levels of 31 % at days 5–7, *P* = 0⋅09; 81 % at days 12–14, *P* = 0⋅03; 49 % at days 20–22, *P* = 0⋅02, compared with the baseline.

This effect persisted for at least one menstrual cycle after the suspension of soy intake, with a maximum of persistence for three menstrual cycles. Furthermore, from the multiple regression analysis of ten women in the second trial^(29)^, the reduction of estradiol in both luteal and follicular phases was positively associated with serum and urinary isoflavone levels but not with individual changes in the intake. In both studies, the lowering of progesterone levels in luteal phase was also significant in the case of soy intake, mean 35 % (*P* = 0⋅002) compared with baseline.

Among the six women in the first clinical trial^(26)^, the intervention with soy also led to a significant reduction in dehydroepiandrosterone sulphate (DHEAS) levels (23 %, *P*  = 0⋅03), an intermediary in estradiol synthesis. In the ten women who participated in the second study^(29)^, there were no significant changes in the levels of luteinizing and follicle-stimulating hormones. The lack of variation in gonadotropins can explain the absence of variation in menstrual cycle. However, soy diet reduced progesterone (45 %, *P* < 0⋅0001) and estradiol levels (23 %, *P* < 0⋅01), compared with baseline. The urinary or serum levels of isoflavones did not affected progesterone levels in the multiple regression analysis. The strength of these studies was the assessment of hormone levels based on the menstrual cycle phase. The limitations of these studies have already been discussed in the previous paragraph.

In another clinical trial already discussed, although no changes in cycle length were found following soy foods intervention in twenty women with a follow-up of at least seven menstrual cycles, a significant reduction in follicular phase by 9⋅3 % (*P* < 0⋅05) in estradiol concentrations was observed, but not in luteal phase^(28)^. Progesterone and sex hormone-binding globulin (SHGB) levels were not significantly changed by soy intake. The reduction of estradiol concentrations observed became statistical marginal (8⋅9 %, *P* = 0⋅06) when analysis was restricted to the ‘clean’ dataset: data after exclusion of thirteen specimens collected too soon or too late after ovulation. From a sub-analysis on ethnic characteristics, it was further highlighted that only Asian women showed a significant reduction in follicular estradiol from baseline (17⋅4 %).

From the analysis of urinary excretion of isoflavones normalised for creatinine during the intervention with soy, Asian women had significantly greater excretion of isoflavones than non-Asian women. However, the difference became not significant after adjustment for isoflavone intake. Isoflavones concentrations did not show significant differences between participants at baseline.

The clinical trial was limited to a small sample size, lacking of control/placebo group and there was no characterisation of equol-competence. However, the work had several strengths: the real evaluation of luteal and follicular phase through the dosage of urinary LH:creatinine ratio, the characterisation of sampling according to the cycle; the evaluation of isoflavone content in foods used for the intervention and quantification of urinary isoflavones to check compliance; the use of soy foods and not supplements or extracts to approach a real-life pattern; the characterisation of diet at various steps of clinical trial to avoid confounding mechanisms; the stratification by ethnicity which indirectly showed the effect on equol-producer individuals.

This latter aspect suggests a differential capacity for metabolising isoflavones even if these differences were no longer significant when corrected for the intake of isoflavones and estradiol levels were not significantly associated with urinary excretion of isoflavones. In addition, no significant changes in progesterone, LH or SHBG were found in the whole study sample. From the sub-analysis by ethnic stratification, follicular SHBG levels were higher in non-Asians.

Previously, Petrakis and colleagues proposed an interventional study with a soy isolate (37⋅4 g of soy protein containing 37⋅4 mg of genistein) on twenty-four women (pre- and post-menopause) followed for 6 months plus 3 months pre-intervention and 3 months post-washout^(25)^. Estradiol levels showed increased plasma concentrations during the intervention period among premenopausal women (*n*: 14) in both luteal and follicular phases (composite menstrual cycle assessment). No changes in progesterone and SHBG concentrations from baseline were observed. Compliance with the intervention was suggested by urinary excretion of isoflavones. While isoflavones and their metabolites were undetectable in the pre-soy phase, during intervention the 24 h output of urinary excretion was 3⋅12 mg for genistein (7⋅4 % of the ingestion). Excretion of daidzein and its metabolites dihydrodaidzein and *O*-desmethylangolensin (36⋅01, 3⋅14 and 2⋅27 mg, respectively) accounted for 42⋅1 % of daidzein ingested. Although this clinical trial showed the long-term effect of soy ingestion on serum hormone levels, it was a pilot study with a limited number of participants (fourteen premenopausal women). Furthermore, diet and energy intake were not investigated and sampling was not well-timed to menstrual cycle. They evaluated the hormonal variations during menstrual cycle through the ‘composite’ construct that considered the cumulative information of the day of menstrual cycle for specimens. The authors defined the unusual estradiol increase as ‘erratic’.

Based on our literature search, we also identified two observational studies: a cross-sectional study published in 1997 by Nagata *et al.*^(27)^ and a longitudinal study published in 2013 by Filiberto *et al.*^(37)^. In the study by Nagata and colleagues, fifty Japanese women were enrolled to evaluate the association between soy intake (using an FFQ) and hormone levels. After adjustments, an inverse correlation between estradiol and soy intake was highlighted on the 22nd day of menstrual cycle (r: −0⋅32, *P* = 0⋅04) but not on the 11th.

SHBG levels were not associated with the intake of soy foods, except in the case of miso intake on the 22nd day of cycle (*r*: −0⋅36, *P* = 0⋅02). Day 22 should correspond to the mid-luteal phase, however, the authors pointed out that participants exhibited different lengths of menstrual cycle and this could have been a source of heterogeneity that was used as a covariate in the regression model. However, soy intake did not correlate with cycle length (*r*: −0⋅12, *P* = 0⋅45). The evaluation at two different times of menstrual cycle allowed to discriminate the effect between luteal and follicular phases but not day by day hormonal fluctuations. However, the number of participants was limited for a cross-sectional study, and dietary survey through frequency questionnaires in the absence of an assessment of blood or urine isoflavone levels could lead to uncertainty. The study's strength include a large consumption of soy and by consistent inter-individual variability among participants (total intake of 37⋅9 ± 26⋅1 g/d), which allows better detection of cross-sectional correlations.

Instead, in the cohort study by Filiberto and colleagues, 259 American women were followed for at least 2 menstrual cycles. Mildly increased levels of SHBG were associated with higher dietary isoflavone intakes (Q4 [1⋅6–78⋅8 mg/d] *v*. Q1 [0⋅0–0⋅3 mg/d]) in the adjusted linear mixed model (*β*: 0⋅09, 95 % CI 0⋅02, 0⋅16), but no correlation was found for estradiol, progesterone, LH, FSH levels or anovulatory events. 44 % of women of Asian descent were in the highest quartile of isoflavone intake. Although isoflavones can be found in many foods, not soy foods can be considered negligible sources of these compounds. The influence on SHBG levels can have a beneficial effect from an endocrine point of view, without negative effects on ovulation. The present study has numerous strengths: a large sample of participants with good adherence to the study, a detailed assessment of dietary habits, and comprehensive sampling during all phases of menstrual cycle. Finally, the authors made a detailed assessment of confounders (diet, ethnicity, age and BMI). However, there were also limitations: the duration of the study which was limited to two menstrual cycles and an evaluation of equol-producers among individuals was lacking. Furthermore, the intake of isoflavones among participants was very low and this made it difficult to compare the findings with clinical trials that often use intakes similar to Asian populations (23–84⋅4 mg/d).

In the previously mentioned meta-analysis by Hooper and colleagues^(59)^, reduction of about 22 % of FSH (SMD: −0⋅45 UI/l, 95 % CI −0⋅79, −0⋅11, *P* = 0⋅01) and of about 4 % of LH (SMD: −0⋅34 IU/l, 95 % CI −0⋅68, −0⋅01, *P* = 0⋅05) were related to the intake of soy or isoflavones. However, levels of progesterone, estradiol, free estradiol, estrone and SHBG did not show significant differences.

These changes may have resulted in the mild, non-clinically relevant prolongation of menstrual cycle, as discussed in the previous section. However, after removing data from studies with elevated bias risk, two studies were included in the sensitivity analysis with a consequent loss of statistical significance for LH levels. Nevertheless, a reduction in FSH levels was confirmed (SMD: −0⋅87 IU/l, 95 % CI −1⋅72, −0⋅02). However, the number of combined participants of the two studies was very limited (*n*: 40).

Steroid hormones (estradiol, progesterone and DHEAS) play a role in epithelial cell proliferation in mammals. The duration of menstrual cycle, especially in luteal phase, can also have a direct influence on the mammary gland proliferation, through a reduction in exposure of the epithelium to proliferative hormones. For these reasons, studies that evaluated the ovarian hormones secretion were aimed at exploring the potential beneficial effect of soy on breast cancer prevention, but they were not designed for the evaluation of endocrine consequences, including fertility.

The reduction of estradiol and progesterone could postpone ovulation by lengthening the menstrual cycle. The effects obtained from selected studies do not seem to show a clear significance regarding fertility and menstrual cycle length, as discussed in the previous paragraph. There is a limited trend in estradiol reduction related to soy consumption; however, in their interventional study, Petrakis and colleagues observed an unusual increase of estradiol levels^(25)^.

In addition, in the work of Kohama and colleagues, an increase in estradiol levels following intervention with soy compared with baseline was shown^(33)^. However, the subjects enrolled were women with secondary amenorrhea and therefore this variation could have a different meaning compared with results discussed in this section, obtained in the healthy population.

There are clues about the association between soy intake and the increase in SHBG levels. It is plausible that isoflavones bind to this blood carrier and stimulate its hepatic synthesis. This could favour the bioavailability of sex hormones^(60)^. However, in the work of Filiberto and colleagues^(37)^, even if the correlation between isoflavones and the increase in SHBG was highlighted, the dosage of estradiol and free estradiol did not show significant correlations, although the estimate of free estradiol was done through Sodergard's formula^(61)^, so a direct dosage would be more reliable.

It is important to evaluate the levels of hormones that fluctuate during the cycle at several points. This could be done by empirically monitoring ovulation to get a real information of menstrual phase, such as quantifying the urinary LH peak as a marker of ovulation, as done by Wu *et al.*^(28)^.

### Polycystic ovary syndrome

Polycystic ovary syndrome (PCOS) is a major endocrine and metabolic disorder in women^(62,63)^. It is an endocrine dysfunction that includes hormonal alterations (increased levels of adrenal and ovarian androgens and SHBG secretion from the liver) and anovulatory disorders^(64)^. These alterations easily lead to hyperandrogenism and irregular menstrual cycles. Furthermore, women with PCOS display a higher prevalence of hyperinsulinemia, dyslipidemia, insulin resistance and obesity compared to healthy population. The disease etiology is still debated but it seems to involve inflammatory mechanisms and oxidative stress^(65,66)^. The diagnosis of PCOS occurs in the presence of at least two of the three Rotterdam Criteria: oligo or anovulation, polycystic ovary morphology and biochemical or clinical hyperandrogenism^(67)^.

Four clinical trials were found among search engines results: two longitudinal pilot studies^(34,46)^ and two interventional studies with a parallel design, both conducted in Iranian populations^(35,43)^.

Romualdi and colleagues in 2008 enrolled twelve Italian women with metabolic syndrome and PCOS and with a follow-up of 6 months using 36 mg/d of oral genistein as an intervention^(34)^. Researchers did not observe any clinical improvement, alteration of menstrual cycle or hormonal alteration (estradiol, SHBG, DHEAS, androstenedione, testosterone, FSH, LH) compared with baseline levels. Patients showed plasma androgens levels above or at the upper limit the normal range, at baseline. Improvements were observed only in lipid profile (circulating total cholesterol, LDL and LDL/HDL ratio and triglycerides). Despite the 6-month duration of the clinical trial, the lack of a placebo group, the absence of characterisation of equol-competence among individuals and the limited number of participants reduced the strength of the results obtained.

The same amount of genistein was used in a parallel clinical trial on 137 Iranian women with PCOS with a 3-month follow-up^(35)^. In the present study, the intervention group showed improvements in hormonal circulating levels compared with baseline, which consisted in the reduction of LH levels (−9⋅4 %, *P* = 0⋅000), testosterone (−5⋅6 %, *P* = 0⋅000) and DHEAS (−8⋅7 %, *P* = 0⋅000), with no significant changes in the control group. FSH levels were not significantly changed after genistein intervention. Genistein treatment reduced LDL cholesterol and triglycerides levels. Sampling involved synchronisation on the third day of menstrual cycle follicular phase, spontaneous or pharmacologically induced. Although the clinical trial was quasi-randomised, with a placebo group, double-blinded, authors did not characterise the dietary regimen of individuals as well as their ability to effectively absorb and metabolise soy isoflavones. Furthermore, hormone levels were evaluated only at baseline, without taking into account the differences between the two groups.

Jamilian and colleagues in 2016 conducted another parallel clinical trial on seventy Iranian women with PCOS, using 50 mg/d of soy isoflavones for a 1-month follow-up^(43)^. From the general linear model of the analysis of covariance, the intervention with soy reduced free androgen index (−0⋅02 ± 0⋅005 *v*. +0⋅01 ± 0⋅005, *P* < 0⋅001), total testosterone (−0⋅1 ± 0⋅05 *v*. +0⋅1 ± 0⋅05 ng/ml, *P* = 0⋅008) and increased SHBG levels (+4⋅0 ± 0⋅8 *v*. −1⋅4 ± 0⋅8 nmol/l, *P* < 0⋅001) compared with placebo (adjusted for baseline values). No significant differences were appreciated for free testosterone and DHEAS. The hormonal improvement has been followed by clinical ameliorations such as the reduction of alopecia, serum insulin levels, HOMA-B (homeostasis model of assessment-B cell function) and HOMA-IR (homeostasis model of assessment-insulin resistance) index among patients in the intervention arm. Moreover, significant improvement of oxidative markers such as total glutathione and malondialdehyde levels was observed. Although this was a randomised, placebo-controlled and double-blinded trial with a sample size appropriate to the power of detection, there was no evaluation of serum and urinary levels of isoflavones and/or metabolites. There was no evaluation of dietary habits and the determination of hormone levels was performed using non-validated ELISA kits, due to limited budget. For these reasons, results should be interpreted with caution.

Recently, Haudum and colleagues conducted a longitudinal case-control clinical trial on forty-four Australian patients (twenty-four PCOS and twenty healthy controls) using 400 ml/d of soy milk (containing approximately 50 mg of isoflavones, 13⋅2 g protein) for a 3-d pilot study^(46)^. The study included the evaluation of patients’ microbiota composition as the primary endpoint, but androgen levels were also evaluated with AMH as markers of fertility as a secondary endpoint.

Major equol production was associated with a reduction in androgens levels (total testosterone, free testosterone and androstenedione), in the whole cohort. However, stratification for the control group or PCOS patients did not show a significant correlation between androgens and equol production. Likewise, equol-producers showed lower AMH levels in the whole cohort as well as in participants in PCOS or control groups. No changes were highlighted for DHEA, DHEAS, dihydrotestosterone (DHT) concentration or LH:FSH ratio. Microbial alpha diversity and glucose homeostasis improved in PCOS group after isoflavone intervention, resembling the control group profile at baseline. This was a short pilot study with a small sample size in subgroups. The power analysis concerning variation in isoflavone urinary excretion accounted for a sample size of 25 for >90 % detection power. Furthermore, the use of spot urine samples could generate an underestimation of urinary isoflavones quantification. Even if the clinical trial did not include a placebo group or randomisation, the presence of a control group and the evaluation of equol-producer individuals mitigated these issues.

From data that emerged on individuals with PCOS, there is no homogeneous improvement effect on hormonal picture, on menstrual cycle and therefore on fertility associated with soy consumption. Overall, a trend toward improvement can be appreciated but further studies are necessary to confirm the beneficial effect. Among the studies already cited, however, we must consider the work of Kohoama and colleagues^(33)^, which showed fertility improvements following intervention with black soy extract in individuals with secondary amenorrhea, including patients with PCOS.

### Assisted reproduction technology

Interestingly, soy often appears in literature as a food with a beneficial effect on fertility, especially in the case of pregnancy search^(68)^. Four papers were found about assisted reproduction technology, two of which were interventional studies by Unifer and colleagues, using high intakes of soy phytoestrogens as adjuvant^(31,32)^. Moreover, two recent observational cohort studies by Chavarro and colleagues evaluated the association between soy consumption and *in vitro* fertilisation outcomes^(40,42)^.

In the first clinical trial by Unifer and colleagues, 1500 mg/d of isoflavones from soy or placebo were administered for 10 d to 134 women who had been infertile for at least 2 years, undergoing intrauterine insemination after 100 mg/d for 5 d of clomiphene citrate treatments (an ovulation inducer)^(31)^. Despite the significant increase in FSH, LH and estradiol in both intervention arms, the endometrial thickness (assessed by transvaginal sonography) had a major improvement in the intervention group compared with placebo. Furthermore, the intervention group showed lower rates of miscarriage (*n*: 2, 3⋅1 % *v*. *n*: 6, 8⋅7 %; *P* < 0⋅05) and higher rates of pregnancy (*n*: 13, 20⋅0 % *v*. *n*: 3, 4⋅4 %; *P* < 0⋅05) compared with placebo. Follicular development, the number of preovulatory follicles and the pulsatility index values were not different between groups after intervention.

Presumably, treatment with pharmacological concentrations of soy phytoestrogens allows mitigating the negative effect of clomiphene citrate on endometrial tissue, thus facilitating embryo implantation.

The same type of soy phytoestrogen intervention was subsequently used by Unifer and colleagues in a second clinical trial on 213 infertile women undergoing *in vitro* fertilisation with embryo transfer cycles after intramuscular progesterone treatments (50 mg/d) with or without (placebo) 1500 mg/d of soy isoflavones intake^(32)^.

The concomitant treatment with soybean phytoestrogens significantly increased the implantation rate (25⋅4 % *v*. 20⋅2 %; *P* < 0⋅05), the pregnancy rate (39⋅3 % *v*. 20⋅9 %; *P* < 0⋅05) and the pregnancy-to-delivery rate (30⋅3 % *v*. 16⋅2 %; *P* < 0⋅05) compared with placebo. No significant differences were found in the spontaneous abortion rate, the number and quality of embryos transferred or oocytes fertilised. Regarding the two mentioned studies, the use of very high amounts of isoflavones is noteworthy because it is not possible to obtain such a dose through diet, therefore the effects found can be interpreted as a pharmacological and not nutritional intervention.

These clinical trials had several strengths including the presence of a placebo group, randomisation, double-blinding and recruitment of a wide number of participants. Moreover, couples with male infertility issues were excluded. The influence of high-dose of isoflavones on fertility emerging from the studies is difficult to be transferred to other groups of individuals with other ethnicity or different treatments.

Regarding the observational studies available, in 2015 Venegas *et al.* recruited 315 USA women underwent 530 cycles of assisted reproduction technology^(40)^. There was a significant correlation between dietary soy consumption and fertilisation rate (77 % *v*. 71 %, *P* = 0⋅004), age-adjusted pregnancy (52 % *v*. 41 %, *P* = 0⋅03) or age-adjusted live birth rate (44 % *v*. 31 %, *P* = 0⋅007) among soy consumers compared with non-consumers. Participants were divided into four categories: non-consumers and tertiles of soy intake. Live birth odds in the multivariable-adjusted analysis was higher among women in the second tertile of soy intake, consuming 2⋅64–7⋅55 mg/d of soy isoflavones (OR: 1⋅87; 95 % CI 1⋅12, 3⋅14) and among women in the third tertile of soy intake, consuming 7⋅56–27⋅89 mg/d of isoflavones (OR: 1⋅77; 95 % CI 1⋅03, 3⋅03) compared with no consumption, but without a significant linear trend. The adjustment for male partner intake of soy in the subgroup analysis did not change the association. As expected, women with the highest soy consumption were more likely to be of Asian descent. Soy consumption was not related to estradiol levels or endometrial thickness. This aspect was different from the results of the clinical trials with high intakes listed above, perhaps due to very different intakes (mean isoflavone consumption of 3⋅4 mg/d in this cohort). Despite the sample size and full follow-up for endpoints evaluation, the study displays limitations. The ethnicity assessment of participants was useful in identifying, as might be expected, a greater consumption of soy foods by Asian individuals. However, ethnicity was not used for outcomes stratification. Furthermore, there was no evaluation of metabolic utilisation capacity of isoflavones and their absorption by measuring serum and urinary levels. This may have influenced the presence of large confidence intervals.

Similar to the previous observational study, Chavarro *et al.* conducted another prospective cohort study on 239 American women undergoing assisted reproductive technology^(42)^. The authors found an association between pregnancy outcomes and urinary Bisphenol A (BPA), dependent on soy consumption in the multivariable-adjusted mixed model. Implantation (*P* for interaction <0⋅02), pregnancy (*P* for interaction <0⋅03) and live birth rates (*P* for interaction <0⋅01) were higher among soy-consumers (*n*: 176, 74 %; mean isoflavone intake of 3⋅4 mg/d) without linear dependence with urinary BPA quartiles (*P* trend >0⋅05), compared with no consumer who had lower rates with higher BPA excretion (*P* trend <0⋅05). This suggests a protective effect of soy against fertility disturbance by BPA. The study did not evaluate circulating or urinary levels of isoflavones to verify the ability to metabolise isoflavones. Furthermore, the use of spot urine samples for BPA quantification may have underestimated its exposure.

While the observational data better reflect the effects of diet in free-living conditions compared with experimental settings of clinical trials, the use of food frequency questionnaires exposes to possible misclassification and measurement errors. Furthermore, the evaluation of dietary pattern before infertility treatments does not exclude the possibility that soy consumption may have been influenced by the search for a healthy pattern to achieve pregnancy.

From obtained data, it seems likely that soy consumption, not only in the form of isoflavones in pharmacological quantities, could have a beneficial effect on fertility, especially in those individuals with fertility problems. However, among fertile individuals, it may have a neutral effect, as discussed in the previous paragraphs.

### Influence of soy in early stages

Although some works investigate the relationship between consumption of soy formulations in infancy and age at menarche, as well as the onset of puberty or pre-puberty reproductive organ size, these outcomes are not strictly related to fertility in reproductive age^(69–71)^. The possibility of a sexual development disorder as a neonatal programming effect is an often raised hypothesis because circulating levels of isoflavones are higher in soy-fed infants compared with cow milk formula or breastfed infants^(69)^.

However, a clear effect on reproductive system has never been highlighted, especially due to the absence of observational studies designed for this purpose. In 2015, a longitudinal study found no differences in sexual organ development at 5 years of age between cow milk formula, breast milk and soy formula feeding^(69)^. Although not strictly related to the aspect of fertility, the study is still ongoing (Clinicaltrials.gov: NCT00616395) intending to follow the participants to evaluate effects on reproductive functions, later in life.

The only study found about the effect of exposure to soy in childhood and reproductive functions is the retrospective study by Strom and colleagues^(30)^. The authors found no significant differences in reproductive outcomes (missed menstrual periods, pregnancy, live births, abortions, miscarriages, full-term deliveries, preterm deliveries, etc.) between 128 women fed with soy-based formula and 268 women fed with cow milk formula during infancy. Longer, not clinically relevant duration of menstrual bleeding (adjusted MD: 0⋅37 d, 95 % CI 0⋅06, 0⋅68), without differences in severity of menstrual flow was observed. However, results are questionable due to the lack of hormone level measurements or reproductive functions. Currently, data are insufficient to assess the effect of early-stage soy exposure on fertility-related outcomes.

Table 2 summarises main limitations about the studies discussed. Only three articles declared power analysis to assess adequate sample size^(30,43,46)^. However, this omission does not necessarily imply that the assessment has not been carried out.

**Table 2.** Limitations of clinical study

Reference	Randomisation	Blinding	Power analysis	Placebo group	Diet characterisation	Adjustment for confounders
Petrakis *et al.*^(25)^	No	No	No	No	No	No
Lu *et al.*^(26)^	No	No	No	No	No	No
Nagata *et al.*^(27)^	–	–	No	–	Yes	Yes
Wu *et al.*^(28)^	No	No	No	No	Yes	No
Lu *et al.*^(29)^	No	No	No	No	Yes	Yes
Strom *et al.*^(30)^	–	–	Yes	–	Partial	Yes
Unfer *et al.*^(31)^	Yes	Yes	No	Yes	No	No
Unfer *et al.*^(32)^	Yes	Yes	No	Yes	No	No
Kohama *et al.*^(33)^	No	No	No	Control	No	No
Romualdi *et al.*^(34)^	No	No	No	No	No	No
Khani *et al.*^(35)^	Partial	Yes	No	Yes	No	No
Jarrell *et al.*^(36)^	–	–	No	–	Partial	No
Filiberto *et al.*^(37)^	–	–	No	–	Yes	Yes
Jacobsen *et al.*^(38)^	–	–	No	–	Yes	Yes
Mumford *et al.*^(39)^	–	–	No	–	No	Yes
Vanegas *et al.*^(40)^	–	–	No	–	Yes	Yes
Andrews *et al.*^(41)^	–	–	No	–	Yes	Yes
Chavarro *et al.*^(42)^	–	–	No	–	Yes	Yes
Jamilian *et al.*^(43)^	Yes	Yes	Yes	Yes	No	Yes
Wesselink *et al.*^(44)^	–	–	No	–	Partial	Yes
Levine *et al.*^(45)^	–	–	No	–	Partial	Yes
Haudum *et al.*^(46)^	No	No	Yes	Control	Partial	No

## Proposed mechanisms of isoflavones action

The chemical structure similarity between soy isoflavones and endogenous estrogens has always stimulated the attention for this class of compounds. However, the mechanisms underlying isoflavones effects on human health are manifold.

The interaction between isoflavones and ERβ estrogen receptor results in a competitive effect which in turn blunts endogenous estrogens action^(72)^, as suggested by estrogenic activity of biochanin A and genistein on BT-474 human breast cancer^(73)^. Isoflavones also bind to ERα receptor, albeit with lower affinity. These mechanisms involve genomic regulation with activation of both receptor's subtypes at 1 μM as seen in 293 human embryonal kidney cells in transient gene expression assay^(74)^.

Isoflavones also show effects that do not imply ERα and ERβ involvement. They can bind G-protein-coupled estrogen receptor 1 (GPR30), with effects driven by both genomic and non-genomic regulation involving different cellular signalling pathways, such as intracellular increase of calcium or NO levels^(75)^, as observed in human endothelial cells after stimulation with equol 100 nM^(76)^. Isoflavone genistein inhibited the enzyme tyrosine kinase in human A431 cell membranes at 0⋅7 μg/ml, a very high, non-physiological concentration^(77)^ and act as a ligand for peroxisome proliferator-activated receptors (PPARs) in cultured astrocytes at 500 nM^(78)^. In addition, equol acts on incretins levels in endocrine L cell line GLUTag cells at concentration ranging from 50 to 300 μM, with long-term metabolic consequences^(79)^.

Phytoestrogens can modulate endogenous hormones at micromolar concentrations by influencing the expression of the enzymes cytochrome P450 19 aromatase (Cyp19), 17β-hydroxysteroid dehydrogenase (17β-HSD) and 3β-hydroxysteroid dehydrogenase (3β-HSD), steroid sulfatases (STS) and sulfotransferases (SULTs), enzymes of steroid biosynthetic pathway^(80–82)^. These enzymes convert estrone and androstenedione into estrogen and testosterone^(83,84)^.

Furthermore, phytoestrogens appear to act on SHBG synthesis by altering mRNA levels in hepatocarinoma human cells treated with genistein 20 μM^(85)^, and modulating the balance between bound and free steroids or competing with endogenous sex hormones for the active site binding of the carrier^(86)^.

Last but not least, soy isoflavones can act through an antioxidant mechanism through the stimulation of enzymes responsible for xenobiotics metabolism and oxidative stress reduction *in vitro* at a range of 5–100 μM^(87)^.

Isoflavones in human plasma are usually low (0⋅4–157 nM) in individuals consuming low-isoflavone diets but in large soy-consumers, such as Asian people, isoflavone concentration can reach up to ~4 μM, with equol reaching up to ~40 nM in low consumers and up to ~2 μM in large soy-consumers^(88)^.

**Fig. 2.**
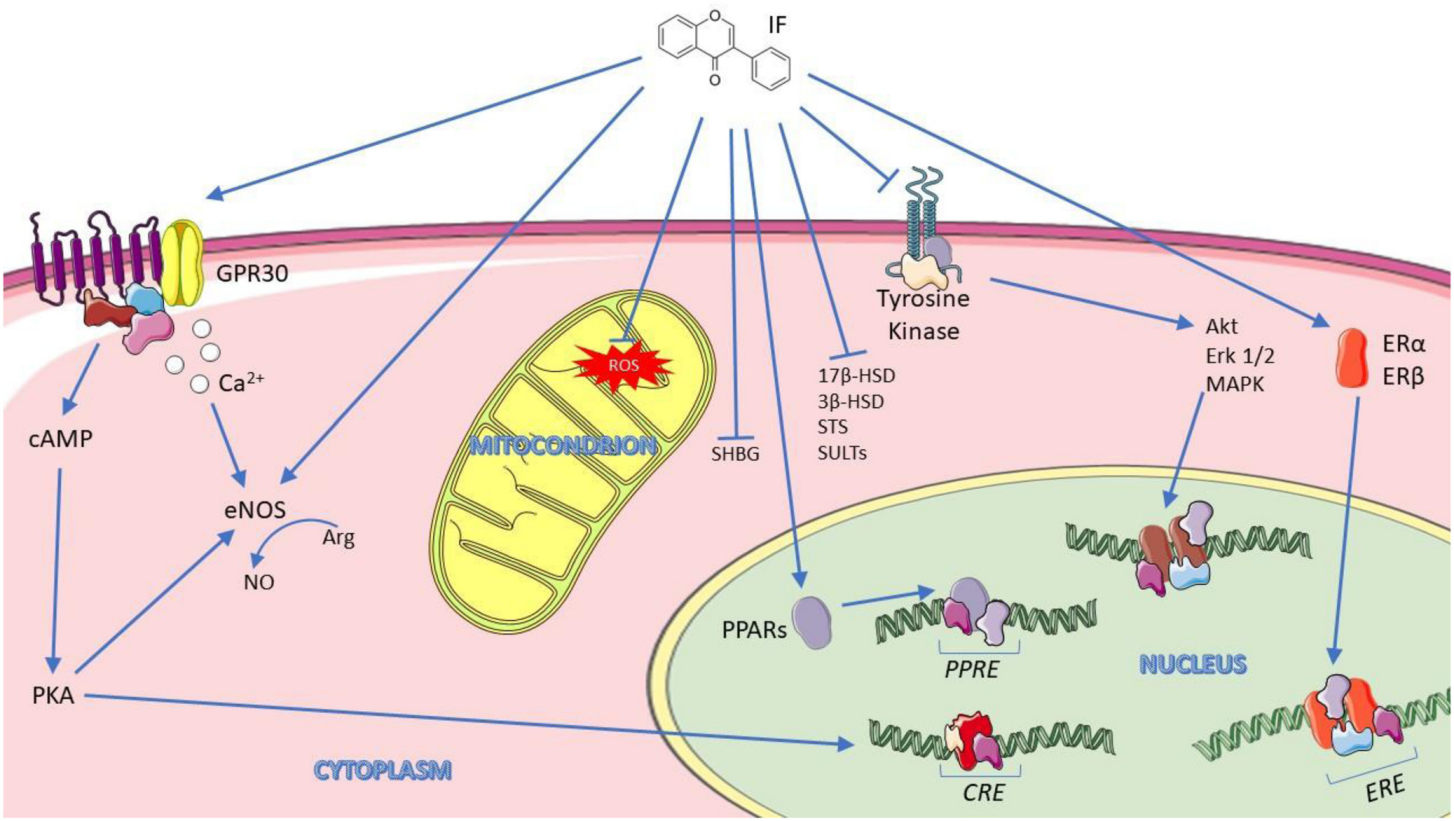
Main cellular mechanism for isoflavones. Adapted from SMART: Servier Medical Art^(89)^.

Fig. 2 highlights the main cellular mechanisms attributed to isoflavones.

## Strengths and limitations

Among the limitations of this review of literature, we must include the possible avoidance of studies that considered fertility as a secondary outcome and which therefore may have escaped from the manuscript selection. In addition, non-English papers could have provided relevant data on the topic, especially those from countries with a more consistent history of soy consumption. However, for articles selection, we used search engines both for scientific literature and specific for clinical trials, without filters application that could have limited the results. Furthermore, the search for sources has been extended to the single manuscripts reference lists. Notably, during manuscript preparation, Messina and colleagues published a technical review on endocrine effect of soy and isoflavones^(90)^. The authors of this recent literature review of available evidence from observational and interventional studies concluded that soy and its components cannot be classified as an endocrine disruptor. These conclusions cover several physiological aspects, including those concerning women's fertility, consistently with the conclusions of this review that highlighted nine additional articles compared with Messina's selection about the topic.

## Discussion and conclusion

Soy isoflavones have repeatedly shown a mild estrogenic effect but at high concentrations they may have enough power to act on hypothalamus and pituitary gland, reducing the ovarian synthesis of estrogens. Furthermore, considering soy as a mere source of isoflavones is extremely reductive. Soy can contain numerous other phytochemicals such as saponins, phytosterols, phytic acid, non-isoflavone flavonoids, peptides, protease inhibitors and other bioactive substances.

In a logical perspective, the effect of soy cannot be attributed to the effect of its isoflavones alone. Furthermore, even at high concentrations, they did not show a clear influence on fertility. This could be due to the scarcity of studies on the topic and the presence of few clinical trials, adequately designed to evaluate soy effect on fertility with consistent timing, with an adequate number of participants, blinding, and randomisation for physiological conditions and the presence of equol-producer individuals.

In particular, among selected studies, only the intervention study by Haudum and colleagues explored the stratification of participants for equol-competence^(46)^. In addition, other studies investigated the urinary concentrations of isoflavones and metabolites, including equol^(39,45)^.

Clinical trials can provide solid causal inferences, but they often have limitations in terms of study duration or intervention design. For example, it should be identified whether the interest is related to pharmacological effect, thus implying the use of high concentrations of soy components, or if the aim is to investigate soy functional effects that can be obtained mimicking eating habits, thus providing soy foods with realistic intake levels. The advantages of observational cohort studies include longer times and wider population samples. Nevertheless, these studies often suffer difficulties in evaluating individual effectiveness as well as in identifying possible confounding factors and population characteristics (ethnicity, health conditions, equol-competence, etc.).

In this context, the evaluation of urine samples cannot be underestimated as a valuable tool for detection of the real bioavailability of isoflavones whose metabolisation requires the intervention of intestinal microbiota. Furthermore, it should be considered that, as already discussed, many studies display several limitations including inadequate sampling of hormone concentrations during all phases of cycle, low number of participants and the lack of a placebo group. These aspects considerably reduce the reliability of results, favouring data misinterpretation. Similarly, the stratification by ethnicity and equol-producers may suggest the nature of interactions between soy and fertility. Even if serum AMH concentrations appear as a useful tool for predicting female fertility, only one study from our selection used them^(46)^. Moreover, difficulties related to data collection about nutritional intakes were available, and individual reporting errors must be taken into account. In particular, information about the adequate choice of updated nutritional tables as well as specific nutritional choices, such as increased soy consumption due to pre-existing socio-cultural and physiological aspects should be collected.

To our knowledge, this is the first comprehensive review on soy effect on women's fertility. While soy appears to have a negligible effect on hormonal network, menstrual cycle length and fertility outcomes of healthy women, some clues emerged from literature on its possible beneficial effect in the case of endocrine diseases such as PCOS. Furthermore, the possible ameliorative influence of soy or its components in the case of assisted reproduction techniques outcomes and pregnancy seeking appears promising and worthy of interest.
